# Effects of nitrogen reduction and aged biochar on soil properties, root characteristics and ascorbic acid glutathione cycle of dryland maize

**DOI:** 10.3389/fpls.2025.1672146

**Published:** 2025-11-27

**Authors:** Chen Sun, Wenhui Ji, Jian Liu, Jianhua Wang, Shuping Hu, Jun Zhou, Ruifang Liu, Yan Li, Jiang Du, Guohui Cao, Zhaoran Wang, Jiying Sun

**Affiliations:** 1 College of Agronomy, Inner Mongolia Agricultural University, Hohhot, China; 2 Vocational and Technical College, Inner Mongolia Agricultural University, Baotou, China; 3 Agricultural and Animal Husbandry Technology Extension Center, Hohhot Agriculture and Animal Husbandry Technology Bureau, Hohhot, China

**Keywords:** nitrogen, aged biochar, soil, root, AsA-GSH cycle, dryland maize

## Abstract

**Introduction:**

Nitrogen (N) and Biochar (B) are well-documented in plant and soil improvement, but their regulatory role in mitigating plant oxidative stress under dryland cultivation is poorly understood. The current study was conducted to investigate the effect of nitrogen reduction and aged biochar on soil texture, root characteristics, antioxidants activities, and yield of maize under dryland cultivation.

**Methods:**

A split plot design was employed in the field experiments, incorporating three nitrogen levels (N0:0, N150:150 and N300:300 kg N•ha^-1^) and four biochar application rates (B0:0, B8:8, B16:16 and B24:24 t•ha⁻¹).

**Results:**

The soil bulk density (SBD) under B24 decreased by 11.07%, however, the soil porosity (SP) increased by 15.11% as compared to B0. The activities of N-acetyl-β-D-glucosaminidase (NAG) and leucine aminopeptidase (LAP) under N150 treatment increased by 42.82% and 17.20% as compared to N300. B8, B16, and B24 treatment significantly increased total root length (TRL) and total root surface area (TRSA) by 5.72-18.65% and 19.12-38.56%, as compared to B0. Maize experienced less oxidative stress under N150 treatment due to the lower accumulation of superoxide radical (O_2_^-^) by 38.32%, and hydrogen peroxide (H_2_O_2_) by 19.25% as compared to N300 treatment. The addition of 24 t•ha^-1^ biochar reduced the levels of O_2_^-^ and H_2_O_2_ by increasing the activities of superoxide dismutase (SOD) by 13.64%, ascorbate peroxidase (APX) 11.86%, monodehydroascorbate reductase (MDHAR) 13.13%, dehydroascorbic reductase (DHAR) 11.50%, and glutathione reductase (GR) 9.82% compared to B0.

**Discussion:**

The results revealed that nitrogen reduction and aged biochar could alleviate the harmful effects of drought stress by improving soil quality, root characteristics, and the enzyme activities in ascorbic acid glutathione (AsA–GSH) cycle of maize which indicate that the nitrogen reduction and aged biochar application might be a sustainable strategy to enhance maize growth under dryland cultivation.

## 1 Introduction

Maize (Zea mays L.) is one of most important cereal crop in China, with a huge planting area and high grain yield (Afreh et al., 2018), yielding over 200 billion metric kilograms per year and accounting for 42.2% of cereal crop yields (Zamora-Re et al., 2020). However, drought stress is one of the most important environmental variables limiting the production potential of maize crop (Muhammad et al., 2022), and the average maize grain yield decreased by drought stress was about 14.70% over the last decade in China (Khan et al., 2019).

The CO_2_ acclimatization rate reduces during drought stress conditions, as a result, the consumption rate becomes lower than the decreasing power rate in the Calvin cycle. A large amount of reactive oxygen species (ROS) is generated as reducing agent molecules are assimilated during the formation of electron transport chains in the photosystem (Grzesiak et al., 2006). The excessive ROS accumulation under drought stress conditions significantly impairs the cell structure such as membrane, proteins, lipids, and nucleic acids, and eventually, resulting in protein denaturation, lipid peroxidation, and DNA disintegration (Abid et al., 2018). Plant antioxidant defense mechanisms encompass enzymatic and non-enzymatic activities that effectively eliminate ROS from cells (Deng et al., 2017; Tewari et al., 2019). The AsA-GSH cycle is an important antioxidant defense system in plants in which antioxidant enzymes synergize with non-enzymatic antioxidants. In this cycle, APX uses AsA to reduce H_2_O_2_ to H_2_O and O_2_, producing Monodehydroascorbate (MDHA) as an intermediate. MDHA is then either directly reduced to AsA by MDHAR or undergoes non-enzymatic disproportionation to form dehydroascorbic acid (DHA). DHA is subsequently reduced back to AsA by MDHAR, while reduced glutathione (GSH) is oxidized to glutathione disulfide (GSSG) in the process. Finally, GSSG is reduced back to GSH by GR (Asthir et al., 2020). This integrated antioxidant system functions through the synergistic interaction of its constituent elements to maintain cellular redox homeostasis (Pandey et al., 2015). However, in severe drought stress conditions, the inherent defense capacity of plants is weakened. Therefore, it is essential to implement an effective agronomic management strategy aimed at enhancing the plant antioxidant defense system, improving drought tolerance, and reducing the production of ROS. Nitrogen is a principal driving factor of soil extracellular enzyme activities, plant physiological and biochemical activities, and plays an essential role during plant growth and production (Liu et al., 2023). Soil extracellular enzymes are protein catalysts produced and secreted by soil microorganisms, playing a crucial role in facilitating biochemical reactions in the soil (Miao et al., 2019). Their primary role is to mediate the decomposition of organic matter and nutrient mineralization, thereby supporting the metabolic demands of plants and microbial communities (Zuccarini et al., 2023). Chen et al. (2018) observed that nitrogen fertilization significantly enhanced the activities of NAG and urease in soil. Furthermore, nitrogen serves as an essential nutrient that critically supports root system development in maize. Previous studies have demonstrated that key root morphological parameters (length, surface area, and volume) exhibit a unimodal response to nitrogen application rates, with optimal values observed at 160 kg·ha^-1^ (Yin et al., 2016). Chen et al. (2015) demonstrated that high nitrogen supply promoted root uptake activity, whereas low nitrogen supply delayed root senescence. The study by Wang et al. (2019b) revealed that low nitrogen supply optimized root system architecture by promoting fine root development and increasing root length density (0.3 cm·cm^-^³), consequently improving both water and nitrogen use efficiency under moderate water stress conditions. Finally, as a fundamental component in protein synthesis, nitrogen significantly influences antioxidant enzyme activity in maize. It has been demonstrated that nitrogen improves the maize capacity to endure drought stress during growth and development phases (Maywald et al., 2022). Previous research showed that applied nitrogen maize outperformed non-applied nitrogen maize in photosynthetic ability, antioxidant enzyme activity, and stress tolerance under various detrimental environmental conditions (Zhang et al., 2007). It has been reported that the application of medium nitrogen fertilizer can make up for the obstacle of carbon metabolism caused by insufficient water (Song et al., 2022), enhance the activities of SOD and APX of maize leaves (Xu et al., 2014). Studies in wheat, soybean, and potato have also shown a significant positive correlation between nitrogen fertilizer and their photosynthetic efficiency and antioxidant metabolism intensity, and a significant negative correlation with the ROS content (Yu et al., 2007).

Biochar, a carbon-rich organic material produced through the slow pyrolysis (<700°C) of biomass under oxygen-limited or anoxic conditions, has gained increasing attention for soil improvement due to its advantageous physicochemical properties, including a highly porous structure, low bulk density, extensive specific surface area, remarkable stability, and exceptional adsorption capacity (Sohi et al., 2010). Studies indicated that biochar amendments significantly decrease soil bulk density while increasing porosity, with field water-holding capacity exhibiting a dose-dependent increase proportional to application rates (Xiao et al., 2016). Zhang et al. (2020) demonstrated that biochar application facilitates the formation of soil aggregate structures and improves their stability under water-deficient conditions, thereby enhancing soil water retention capacity. The enhancement of soil physical properties establishes a favorable substrate environment that promotes microbial development and activity. Research has reported that biochar amendment can modify microbial community structure, increase soil microbial diversity and metabolic activity, thereby upregulating extracellular enzyme activity and accelerating soil nutrient cycling processes (Jin et al., 2024). It has been reported that biochar application significantly increase the activity of key nitrogen-metabolizing enzymes (urease, nitrate reductase, NAG, and LAP), thereby enhancing soil nitrogen cycling processes to meet microbial and plant nitrogen demands (Sun et al., 2022; Shi et al., 2025). Biochar-mediated improvements in soil physical structure and nitrogen cycling processes collectively establish favorable edaphic conditions for root development. It has been reported that biochar amendment significantly enhanced rice root system development through increased primary root length and volume, expanded total and active absorption areas, and moderated senescence processes (Liu et al., 2021). Backer et al. (2017) further demonstrated that biochar application significantly increased total root length, root surface area, and active absorption area of maize, which consequently improved root nitrogen uptake efficiency and kernel nitrogen accumulation. In addition, studies have demonstrated that biochar combined with phosphorus fertilizer significantly increased the activities of SOD, APX, and GR in rice leaves, thereby enhancing the plant’s antioxidant defense system under high-temperature stress conditions (Bamagoos et al., 2021).

Studies have shown that biochar can improve plant growth either by its direct or indirect mechanism of actions. The direct growth-promoting effects of biochar arise from its release of plant-essential nutrients, i.e. K, Ca, Na, Mg, P and S etc., whereas, indirect mechanism involves improving soil physical, chemical and biological characteristics (Cheng et al., 2012; Enders et al., 2012). Although extensive field and controlled studies have consistently demonstrated yield improvements following biochar application (Van Zwieten et al., 2009; Graber et al., 2010; Jeffery et al., 2011), the underlying mechanisms governing these effects remain incompletely understood. In particular, the mechanism of nitrogen reduction and aged biochar application on soil improvement, nutrient metabolism, root development, and scavenging of superoxide radicals and hydrogen peroxide through the ascorbate-glutathione pathway under drought conditions remain poorly understood.

Therefore, the study was conducted to assess the effects of nitrogen reduction and aged biochar application on soil physical properties (bulk density and porosity), nutrient-related enzyme activities (NAG and LAP), root morphology (root length and root surface area), ROS level (O_2_^−^ and H_2_O_2_), and antioxidant enzyme activities (APX, MDHAR, DHAR, and GR) and non-enzymatic antioxidant contents (AsA and GSH) of AsA-GSH cycle under dryland conditions, and quantify the contributions of soil factors, nutrient metabolism, root characteristics, and the ascorbate-glutathione cycle to the formation of dryland maize yield under different nitrogen-biochar regimes, with a view to providing a theoretical basis and data support for the application of nitrogen fertilizer and biochar in dryland maize cultivation.

## 2 Materials and methods

### 2.1 Description of research location

**Figure 1:**
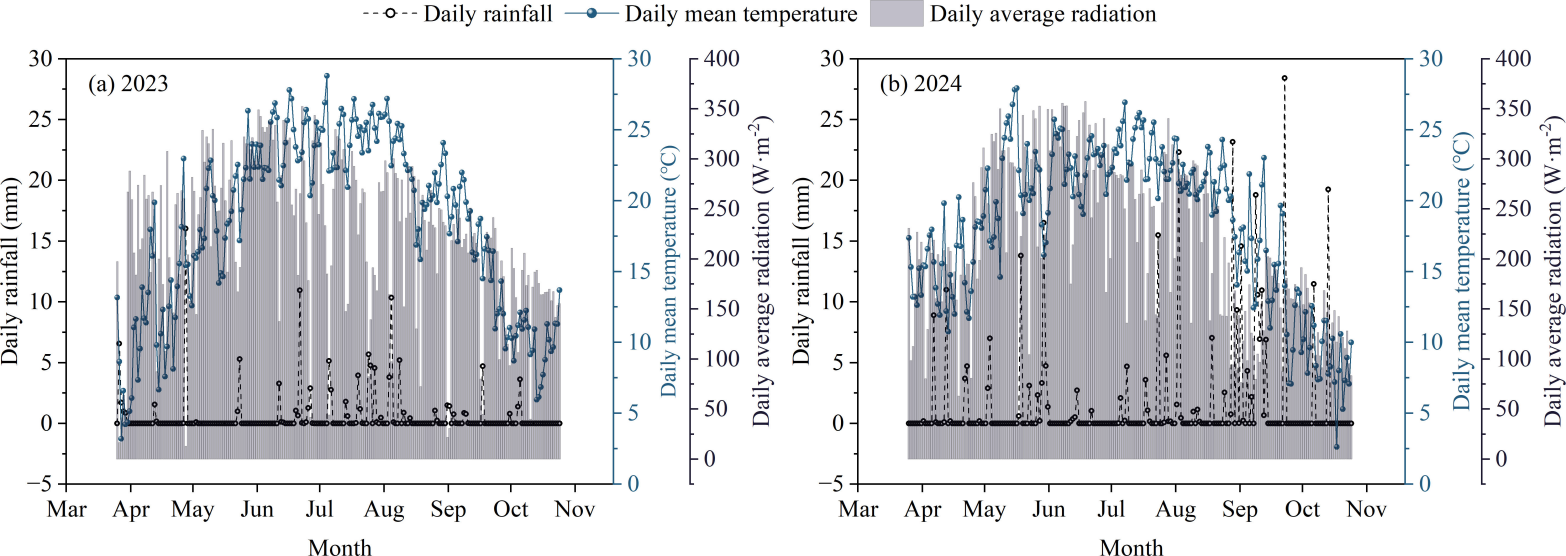
The daily variation of meteorological elements in the test site in 2023 and 2024 [Supplment: **(A)** Changes in meteorological elements in 2023; **(B)** Changes in meteorological elements in 2024].

Two field experiments were performed at the Maize Research Farm of Inner Mongolia Agricultural University, Baotou, Inner Mongolia, China (40°33′ N, 110°31′ E) from 2023 to 2024. The region features a temperate continental climate with a single growing season spanning from May to September. During the two-year growing seasons, precipitation amounted to only 187.22 mm, suggesting pronounced water-limited conditions, whereas mean daily radiation and average temperature were 236.49 W·m^−^² and 21.24°C, respectively (**Figure 1**).

### 2.2 Preparation of biochar

Biochar used in the experiment was obtained by pyrolysis of maize straw at a high temperature of 600 °C for 6 hour produced by Hubei Jinzhi Eco-Energy Co., Ltd. Basic chemical properties of soil and biochar are shown in **Table 1**. The carbon (C), hydrogen (H), nitrogen (N) and oxygen (O) contents of biochar were measured using an elemental analyzer (Elementar, Germany). Biochar was fully applied prior to planting in 2017 and no additional applications were made subsequently.

**Table 1:** Some physical and chemical characteristics of the experimental soil and biochar.

Properties of biochar	Value	Properties of soil	2023	2024
C (%)	47.15	Texture	sandy loam	sandy loam
H (%)	1.54	TN (mg·kg^-1^)	46.61	48.51
O (%)	12.43	AP (mg·kg^-1^)	3.75	3.89
N (%)	0.62	AK (mg·kg^-1^)	83.39	84.83
Ash (%)	19.78	SOC (g·kg^-1^)	17.13	17.6
pH	8.14	pH	7.84	8.01
CEC (cmol·kg^-1^)	20.42	CEC (cmol·kg^-1^)	13.24	14.11

TN, Total nitrogen; AP, Available phosphorus; AK, Available potassium, and SOC, Soil organic carbon; C, Carbon; H, Hydrogen; O, Oxygen; N, Nitrogen; and CEC, Cation exchange capacity.

### 2.3 Experimental design

The field experiments were arranged in a split-plot design with three replications. Nitrogen fertilizer were appointed in main plot, and biochar were allotted to the subplot. Urea was used as an nitrogen source at three different rates: as no nitrogen fertilizer (N0), 150 kg N ha^-1^ (N150), and 300 kg N ha^-1^ (N300). The four biochar levels including 0 t**·**ha^-1^ (B0), 8 t**·**ha^-1^ (B8), 16 t**·**ha^-1^ (B16) and 24 t**·**ha^-1^ (B24). Each treated plot area was 6 m×5 m (length × width). The 12 treatments used in the study were B0N0, B8N0, B16N0, B24N0, B0N150, B8N150, B16N150, B24N150, B0N300, B8N300, B16N300, and B24N300.

The maize cultivar, namely Dika159, was selected as experimental materials and was sown on May (2nd) and May (1st) with a row-row spacing of 60cm and plant-plant spacing of 20cm and harvested on October (4th) and October (2nd) in 2023 and 2024. The planting density was 82,500 plants**·**ha^-1^. Before planting, the recommended basal dosages of calcium magnesium phosphate (18% P_2_O_5_) and potassium chloride (60% K_2_O) were in the soil at a rate of 105 kg·ha^-1^ and 35 kg·ha^-1^. Urea fertilizer was applied three-tenths during the jointing stage and seven-tenths during the large trumpet period. Other field management practices such as tillage practices, weeding, deworming and type and amount of bottom fertilizer and herbicides were consistently performed in all the plots.

### 2.4 Determination of soil samples

Soil samples were obtained during the silking stage (R1) by randomly collecting six 100-cm^3^ soil cores from each plot at a depth of 20 cm using a ring knife (The volume is 100 cm^3^). Three of these cores were air-dried at ambient temperature (22–28°C) for one week to determine soil bulk density and porosity. The other cores were manually homogenized to form a composite sample. The fresh soil was sieved through a 2-mm mesh to eliminate coarse particles and then air-dried at ambient temperature (22–28°C) for one week before assessing N-acetyl-β-D-glucosaminidase (NAG) and leucine aminopeptidase (LAP) activities.

Soil N-acetyl-β-D-glucosaminidase (NAG) activity was measured using a colorimetric 96-well microplate assay, based on changes in substrate concentration as outlined by Liu et al (Liu et al., 2022). Briefly, 0.02 g of air-dried soil was mixed with 10 μl toluene to create a soil suspension. Next, 130 μl of 4-nitrophenol-β-N-acetylglucosamine was added to the suspension, with an equal volume of distilled water serving as the control. Citrate phosphate buffer (pH 6.0) was then added and thoroughly mixed. The mixture was incubated at 37°C for 1 hour, followed by a 5-minute incubation at 90°C. After centrifugation at 25°C for 10 minutes, 130 μl of 0.1 mM Na_2_CO_3_ was added to the supernatant and allowed to react for 2 minutes. Fluorescence was quantified at 400 nm using a microplate fluorometer (Spectramax 190, USA).

Soil leucine aminopeptidase (LAP) activity was assessed by transferring 0.05 g of air-dried soil into 2-ml centrifuge tubes and mixing it with Tris-HCl buffer (pH 7.2). The reaction was initiated by adding 0.05 mM leucyl p-nitroanilide as the substrate, followed by incubation at 37°C for 60 minutes. The mixture was then centrifuged at 8000 rpm for 10 minutes at 4°C, and the supernatant was analyzed spectrophotometrically at 405 nm to determine the reaction product (Wang et al., 2019a).

### 2.5 Determination of root characteristics of maize

At the silking stage (R1), three maize plants exhibiting uniform growth were randomly selected from each treatment. A soil block measuring 40 × 28 × 30 cm (length × width × height) was then extracted from the root zone of each plant using an excavation sampler. The collected soil blocks were gently shaken to dislodge adhering soil particles, transferred to mesh screen bags, and rinsed with a pressurized water spray to eliminate residual soil. Root systems were carefully positioned on the imaging plane for digital capture. The acquired images were subsequently analyzed using WinRhizo ProVision 5.0a (Regent Instruments, Canada) to quantify root morphological parameters, including total length and surface area.

### 2.6 Determination of antioxidant enzymes activities in maize leaves

Three representative fresh maize leaves from each subplot were collected at the silking growth stage (R1) and treated with low temperature liquid N_2_ for preservation.

Hydrogen peroxide (H_2_O_2_) concentration was determined following the method of Velikova et al. (2000). Briefly, approximately 1 g of plant tissue was homogenized in 5 mL of 0.1% trichloroacetic acid (TCA) and centrifuged for 15 min. Subsequently, 0.5 mL of the supernatant was mixed with 0.5 mL of potassium phosphate buffer (10 mM, pH 7.0) and 1 mL of 1 M potassium iodide (KI). Absorbance was measured at 390 nm.

The levels of superoxide radicals (O_2_^-^) in maize leaves was assessed following Elstner and Heupel (1976) and Richmond et al. (1982). Approximately 0.5 g of fresh leaf tissue was homogenized in 5 mL of 65 mM sodium phosphate buffer (pH 7.8) under ice-cold conditions, and the homogenate was centrifuged at 12,000 × g for 20 min at 4°C. The supernatant was used as the enzyme extract. The reaction mixture (final volume 2.0 mL) consisted of 1.0 mL of phosphate buffer (65 mM, pH 7.8), 0.1 mL of 7.5 mM xanthine, 0.1 mL of 10 mM hydroxylammonium chloride, 0.1 mL of the enzyme extract, and 0.4 mL of distilled water. The reaction was initiated by adding 0.3 mL of xanthine oxidase (equivalent to ~60 μg protein) and incubated at 25°C for 20 min. Following incubation, 0.5 mL of the reaction mixture was combined with 0.5 mL of 19 mM sulfanilic acid and 0.5 mL of 1% α-naphthylamine. The solution was mixed thoroughly, allowed to stand for 20 min at room temperature, and absorbance was measured at 530 nm using a UV–Visible spectrophotometer (GENESYS 180, Thermo Fisher Scientific, Massachusetts, USA). Superoxide radical levels were determined from nitrite formation.

Ascorbic acid (ASA) contents was assayed according to Kampfenkel et al. (1995), using three maize leaf samples. Samples (0.5 g) were homogenized in 20 mL of 50 g·L^-1^ trichloroacetic acid (TCA) on ice and brought to 100 mL with TCA. After 10 min extraction, the mixture was filtered and the filtrate collected. For determination, 1 mL of extract was combined with 1 mL TCA (50 g·L^-1^), 1 mL ethanol, 0.5 mL 0.4% phosphoric acid–ethanol, 1 mL 5 g·L^-1^ BP–ethanol, and 0.5 mL 0.3 g·L^-1^ FeCl_3_–ethanol. The mixture was incubated at 30°C for 60 min, and absorbance was recorded at 534 nm. AsA content was calculated from a standard curve.

The contents of reduced glutathione (GSH) and oxidized glutathione (GSSG) were quantified using the method described by Smith (1985). 1 g fresh maize leaf samples were homogenized in 5ml 5% (w/v) pre-cooled sulfosalicylic acid solution and centrifuged at 10,000 rpm for 20 minutes. A 1 mL aliquot of the supernatant was neutralized with 1.5 mL of 0.5 mol·L^-1^ potassium phosphate buffer (pH 7.5) and used for the determination of total glutathione (GSH + GSSG). To determine GSSG exclusively, an additional 1 mL of the neutralized supernatant was incubated with 0.2 mL of 2-vinylpyridine at 25°C for 1.5 hours, which selectively inactivated GSH. Both samples were then extracted twice using 5 mL diethyl ether. The reaction mixture for the assay consisted of the following components: 0.5 mL of 0.1 mol·L^-1^ sodium phosphate buffer (pH 7.5, containing 5 mmol·L^-1^ EDTA), 0.2 mL of 6 mmol·L^-1^ 5,5’-dithiobis-(2-nitrobenzoic acid) (DTNB), 0.1 mL of 2 mmol·L^-1^ NADPH, 0.1 mL (1 unit) of glutathione reductase (GR type III, Sigma Chemical Co.), and 0.1 mL of the sample extract. Absorbance changes at 412 nm were monitored at 25°C. A standard curve was generated using GSH standards to enable accurate quantification.

All enzymatic assays were conducted using the same crude extract. The extraction procedures were consistently performed at 4°C to ensure sample stability and prevent enzyme degradation. The samples were ground in liquid nitrogen and homogenized for 10 minutes in 50 mM sodium phosphate buffer (pH 7.0) supplemented with 0.2 mM ethylenediaminetetraacetic acid (EDTA) and 20% polyvinylpolypyrrolidone (PVPP). The resulting homogenates were filtered and centrifuged at 15000 rmp for 20 minutes at 4°C. The supernatant was subsequently desalted using a Sephadex G-50 column, following the method described by Helmerhorst and Stokes (1980).

Ascorbate peroxidase (APX) activity was determined by monitoring the decrease in absorbance at 290 nm, resulting from the oxidation of ascorbic acid (ASA) over a period of 5–10 minutes, as described by Nakano and Asada (1981). The reaction mixture consisted of 1 mL of 0.68 mmol·L^-1^ ascorbic acid (ASA) and 0.1 mmol·L^-1^ ethylenediaminetetraacetic acid (EDTA) in 0.1 mol·L^-1^ sodium phosphate buffer (pH 7.0), 1 mL of 4 mmol·L^-1^ hydrogen peroxide (H_2_O_2_), and 50–100 µL enzyme extract.

Monodehydroascorbate reductase (MDHAR) activity was assayed following the method described by Zhang and Kirkham (1996). The reaction mixture comprised 50 mmol·L^-1^ sodium phosphate buffer (pH 7.6), 2.5 mmol·L^-1^ ascorbic acid (ASA), 4 units of ASA oxidase, 50 µL of enzyme extract, and 0.1 mmol·L^-1^ nicotinamide adenine dinucleotide (NADH). The oxidation of NADH was monitored by measuring the absorbance at 340 nm.

Dehydroascorbate reductase activity (DHAR) was determined using the method described by Arrigoni et al. (1992). The assay mixture consisted of 0.9 mL of 0.05 mol·L^-1^ potassium phosphate buffer (pH 6.3), 100 μL of 13.5 mmol·L^-1^ reduced glutathione (GSH), 100 μL of 13.5 mmol·L^-1^ dehydroascorbate (DHA), and 100–200 μL of enzyme extract. The enzymatic activity was quantified by measuring the production of ascorbate at a wavelength of 265 nm over a 5-minute period.

Glutathione reductase (GR) activity was measured using the method outlined by Grill (1978), which involved monitoring the oxidation rate of nicotinamide adenine dinucleotide (NADH) at a wavelength of 340 nm. The reaction mixture comprised 0.5 mmol·L^-1^ NADPH, 10 mmol·L^-1^ oxidized glutathione (GSSG), 10 mmol·L^-1^ ethylenediaminetetraacetic acid (EDTA) dissolved in 0.1 mol·L^-1^ sodium phosphate buffer (pH 7.8), and 50–100 μL of enzyme extract.

### 2.7 Grain yield

At the R6 growth stage of maize, an area of 3 m^2^ was randomly selected in each plot, and the grain yield was determined after maize drying and threshing (Yang et al., 2022).


$$\begin{array}{c} Grain\;yield\;(kg\cdot ha^{-1})=maize\;grain\;weight\;(kg\cdot m^{-2})\times 10000\;(m^{2}) \end{array}$$

### 2.8 Statistical analysis

The experiment data were calculated using Excel 2023 (Microsoft, Inc., Redmond WA, USA). The data were analyzed using SPSS version 22.0 (SPSS, Chicago, IL, USA) for Least-Significant Difference test, Duncan’s multiple range test, and correlation analysis. Two-way ANOVA was performed by SAS 9.4 (SAS Institute Inc., Raleigh, CA, USA). Path analysis was implemented by R version 4.4.3. Significance testing was performed using the Duncan’s multiple range test and Least-Significant Difference at a significance level of 5%. All data were expressed as means and standard errors. All graphs were constructed using Origin Pro, version 2024b (Origin Lab Corporation, Northampton, MA, USA).

## 3 Results

### 3.1 Response of soil characteristics and physiological characteristics of maize to different nitrogen levels

**Figure 2:**
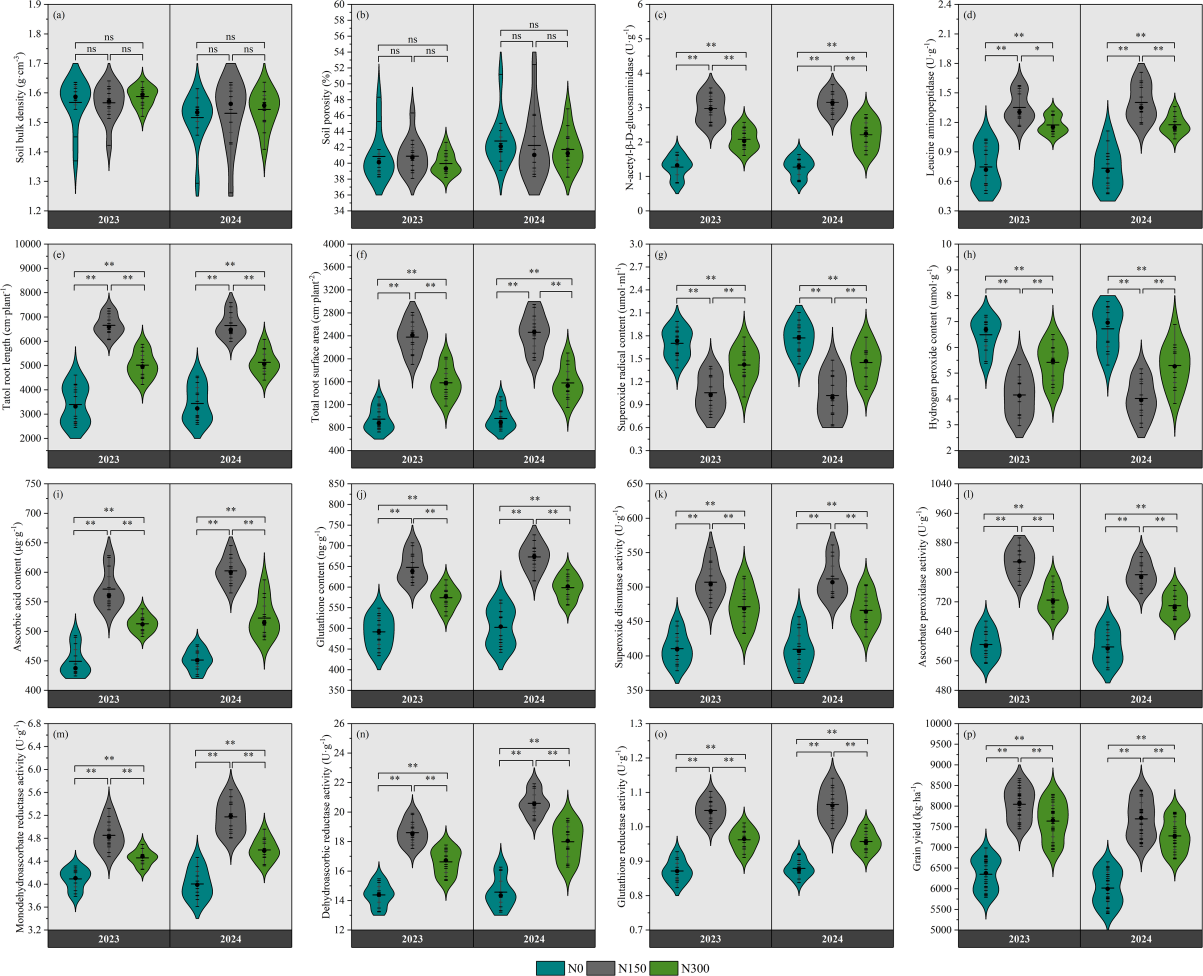
Effects of nitrogen fertilizer on soil bulk density **(A)**, soil porosity **(B)**, N-acetyl-β-D-glucosaminidase activity **(C)**, leucine aminopeptidase activity **(D)**, total root length **(E)**, total root surface area **(F)**, superoxide radical content **(G)**, hydrogen peroxide content **(H)**, ascorbic acid content **(I)**, glutathione content **(J)**, superoxide dismutase **(K)**, ascorbate peroxidase activity **(L)**, monodehydroascorbate activity **(M)**, dehydroascorbic reductase activity **(N)**, glutathione reductase activity **(O)**, and grain yield **(P)**.

**Figure 2** presents the responses of soil physical properties, biological properties, root characteristics, antioxidant enzyme activity, non-enzymatic antioxidant content, ROS levels, and grain yield to different nitrogen levels. No significant variations were observed in soil bulk density or porosity across nitrogen treatments (p>0.05; **Figures 2A, B**), whereas both N-acetyl-β-D-glucosaminidase and leucine aminopeptidase activities exhibited statistically significant differences (p < 0.05; **Figures 2C, D**). Compared to the N0 and N300, the N150 treatment significantly increased total root length by 96.27% and 32.72% (P < 0.01; **Figure 2E**), and total root surface area by 151.10% and 50.45% (P<0.01; **Figure 2F**), respectively. The activities of superoxide dismutase, ascorbate peroxidase, and glutathione reductase under N150 and N300 significantly increased by 23.52% and 14.83%, 37.05% and 19.88%, and 20.16% and 10.39% (P < 0.01; **Figures 2K, L, O**), the content of superoxide radical and hydrogen peroxide significantly decreased by 61.20% and 20.00%, and 56.20% and 19.78% (P < 0.01; **Figures 2G, H**), and grain yield significantly increased by 26.73% and 20.29% (P < 0.01; **Figure 2P**), compared to N0.

### 3.2 Variance analysis of soil and root characteristics

As shown in **Table 2**, significant effects on SBD and SP were observed with biochar application (P < 0.001), whereas no significant effects were detected from nitrogen fertilization (P>0.05). Both nitrogen fertilizer and biochar, as well as their interactions, significantly affected the activities of NAG and LAP, along with TRL and TRSA in both years (P<0.05).

**Table 2:** Variance analysis of soil physical, biological and root characteristics.

Year	Source	SBD (g·cm^-3^)	SP (%)	NAG (U·g^-1^)	LAP (U·g^-1^)	TRL (cm)	TRSA (cm^2^)
2023	Nitrogen	ns	ns	***	***	***	***
	error 1	0.00	2.20	0.01	0.00	52475.86	9870.93
	Biochar	***	***	***	***	***	***
	Nitrogen × Biochar	ns	ns	**	**	***	*
	error 2	0.00	1.28	0.00	0.00	17307.82	6764.92
2024	Nitrogen	ns	ns	***	***	***	***
	error 1	0.00	3.66	0.01	0.01	23771.13	15259.50
	Biochar	***	***	***	***	***	***
	Nitrogen × Biochar	ns	ns	*	**	**	*
	error 2	0.00	4.46	0.01	0.00	31160.59	7766.84

ns, not significant; ^⁎^significant at P < 0.05, ^⁎⁎^significant at P < 0.01 and ^⁎⁎⁎^significant at P < 0.001. Error 1 is main plot error; error 2 is total error, numbers are mean square values. SBD, soil bulk density; SP, soil porosity; NAG, N-acetyl-β-D-glucosaminidase; LAP, leucine aminopeptidase; TRL, total root length; TRSA, total root surface area.

### 3.3 Effects of aged biochar on soil physical properties

**Figure 3:**
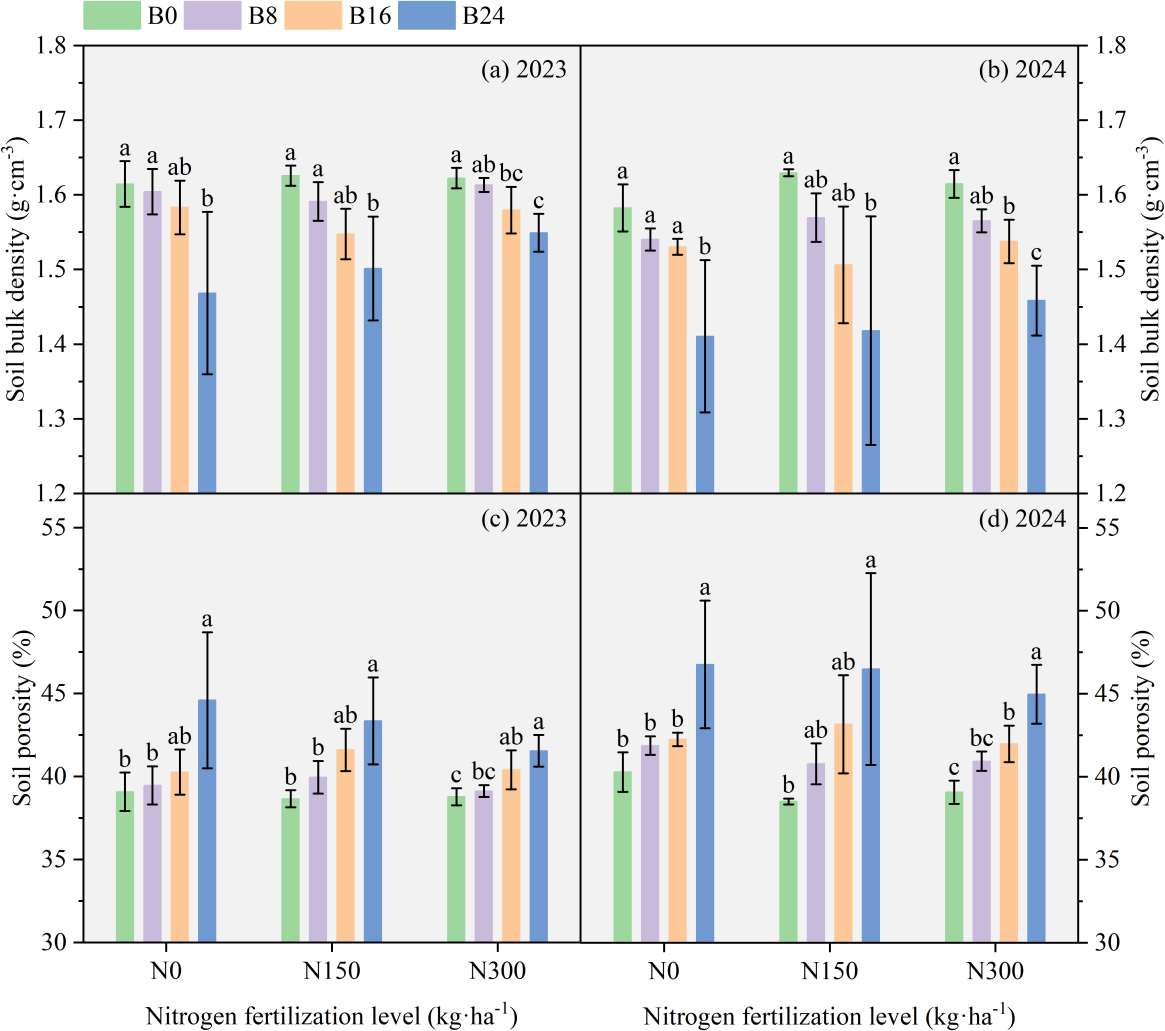
Effect of aged biochar on soil bulk density and porosity. **(A)** soil bulk density in 2023, **(B)** soil bulk density in 2024; **(C)** soil porosity in 2023; **(D)** soil porosity in 2024.

As shown in **Figure 3**, compared with B0, soil bulk density under B8, B16, and B24 decreased by 0.64%, 1.98%, and 9.95% in 2023, and 2.74%, 3.41%, and 12.19% in 2024; soil porosity increased by 0.99%, 3.03%, and 14.11% in 2023, and 3.96%, 4.89%, and 16.11% in 2024, respectively. These results demonstrate that biochar-induced reductions in soil bulk density and enhanced porosity collectively improve soil physical conditions, thereby creating a more favorable environment for both microbial activity and maize root system development.

### 3.4 Effects of nitrogen fertilizer and aged biochar on soil nitrogen metabolism enzyme activities

**Figure 4:**
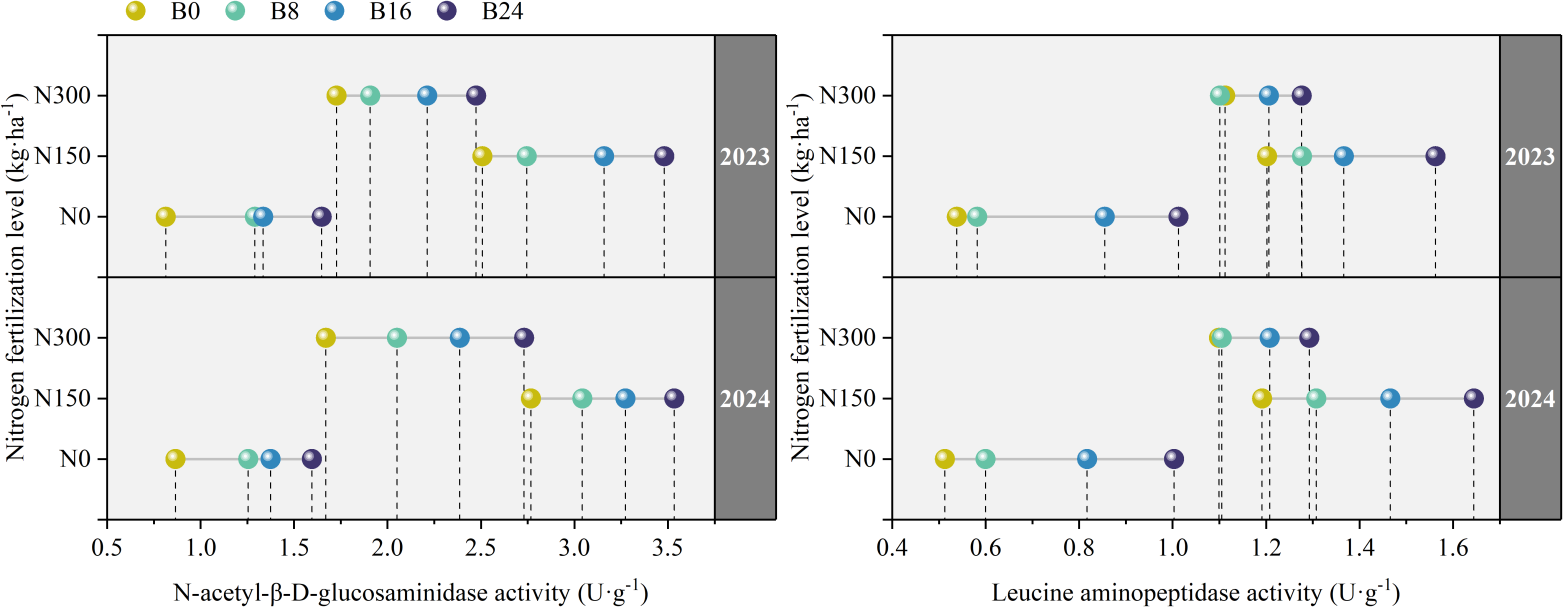
Effects of nitrogen fertilizer and aged biochar on soil NAG and LAP activities.

As shown in **Figure 4**, compared to N0, N150 and N300 significantly increased the activities of NAG and LAP by 133.74% and 63.56%, and 81.02% and 57.18% in 2023, and by 147.89% and 73.66%, and 91.27% and 60.42% in 2024. This indicates that nitrogen application significantly stimulates soil microbial activity and up-regulates key nitrogen-cycling enzymes, demonstrating enhanced soil nitrogen transformation. At the N150 level, compared to B0 treatment, the B8, B16, and B24 treatments significantly increased the activities of NAG and LAP by 9.45%, 25.97%, and 38.79%, and 6.23%, 13.67%, and 29.99% in 2023, and by 9.96%, 18.28%, and 27.72%, and 9.74%, 23.04%, and 38.05% in 2024; respectively. This further indicates that biochar soil amendment promotes microbial-mediated nitrogen conversion and utilization, which in turn facilitates nitrogen uptake by maize roots through improved nutrient availability.

### 3.5 Effects of nitrogen fertilizer and aged biochar on root characteristics

**Figure 5:**
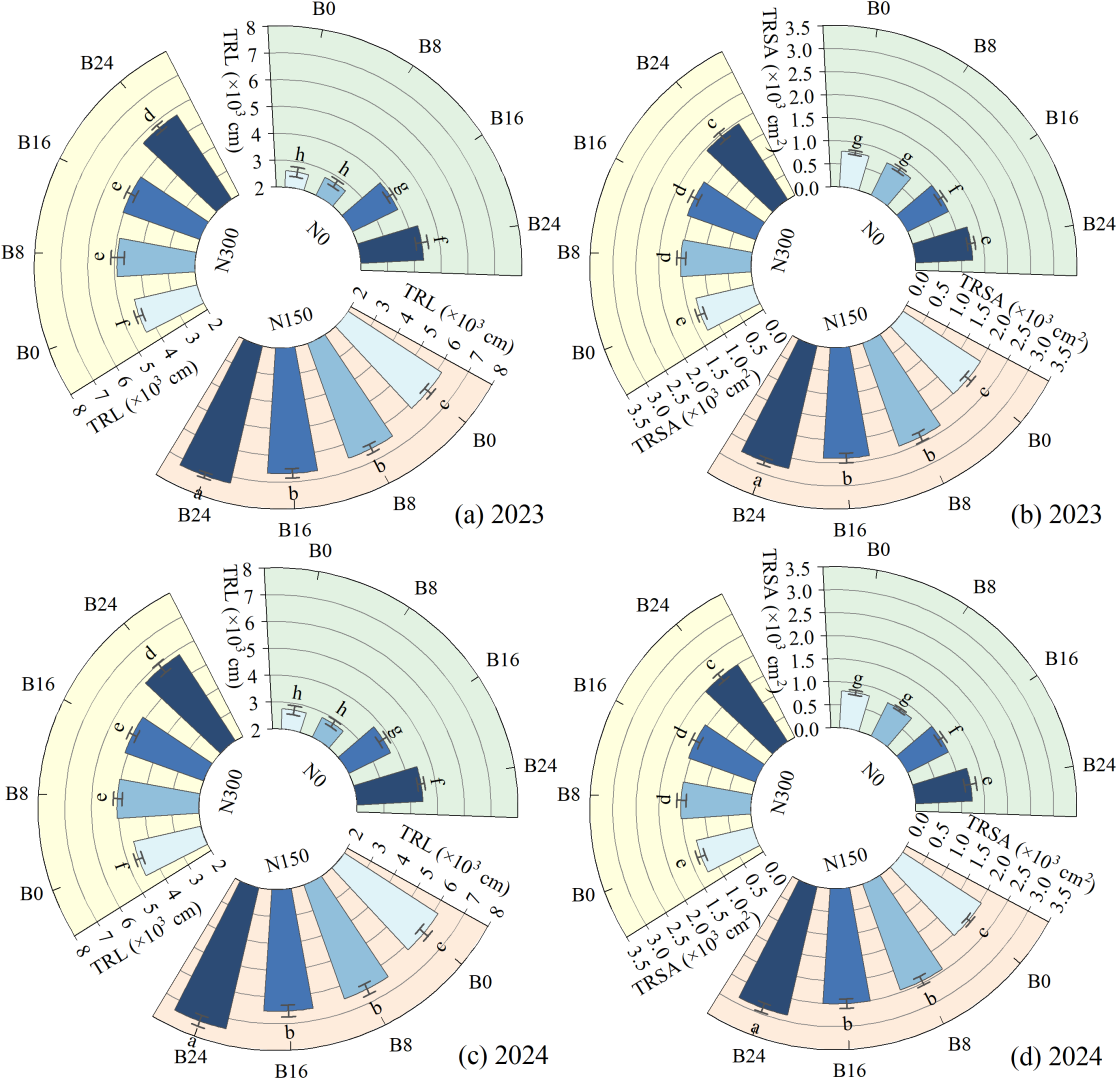
Effects of nitrogen fertilizer and aged biochar on root length and surface area. **(A)** total root length in 2023, **(B)** total root surface area in 2023; **(C)** total root length in 2024, **(D)** total root surface area in 2024; TRL, total root length; TRSA, total root surface area.

As shown in **Figure 5**, compared to N0, N150 and N300 significantly increased TRL and TRSA by 96.27% and 47.89%, and 151.10% and 66.90% in 2023, and by 93.38% and 49.24%, and 156.49% and 64.56% in 2024. This indicates that nitrogen reduction significantly increases root length and surface area in dryland maize, thereby enhancing nutrient and water acquisition capacity. At the N150 level, compared to B0 treatment, the B8, B16, and B24 treatments significantly increased TRL and TRSA by 5.97%, 8.29%, and 17.00%, and 17.95%, 19.49%, and 36.00% in 2023, and by 5.47%, 5.99%, and 20.00%, and 20.28%, 23.23%, and 41.00% in 2024; respectively. These findings further demonstrate that biochar-mediated improvement of soil physical properties establishes favorable edaphic conditions for bidirectional maize root development.

### 3.6 Effect of nitrogen fertilizer and aged biochar on ROS level

**Figure 6:**
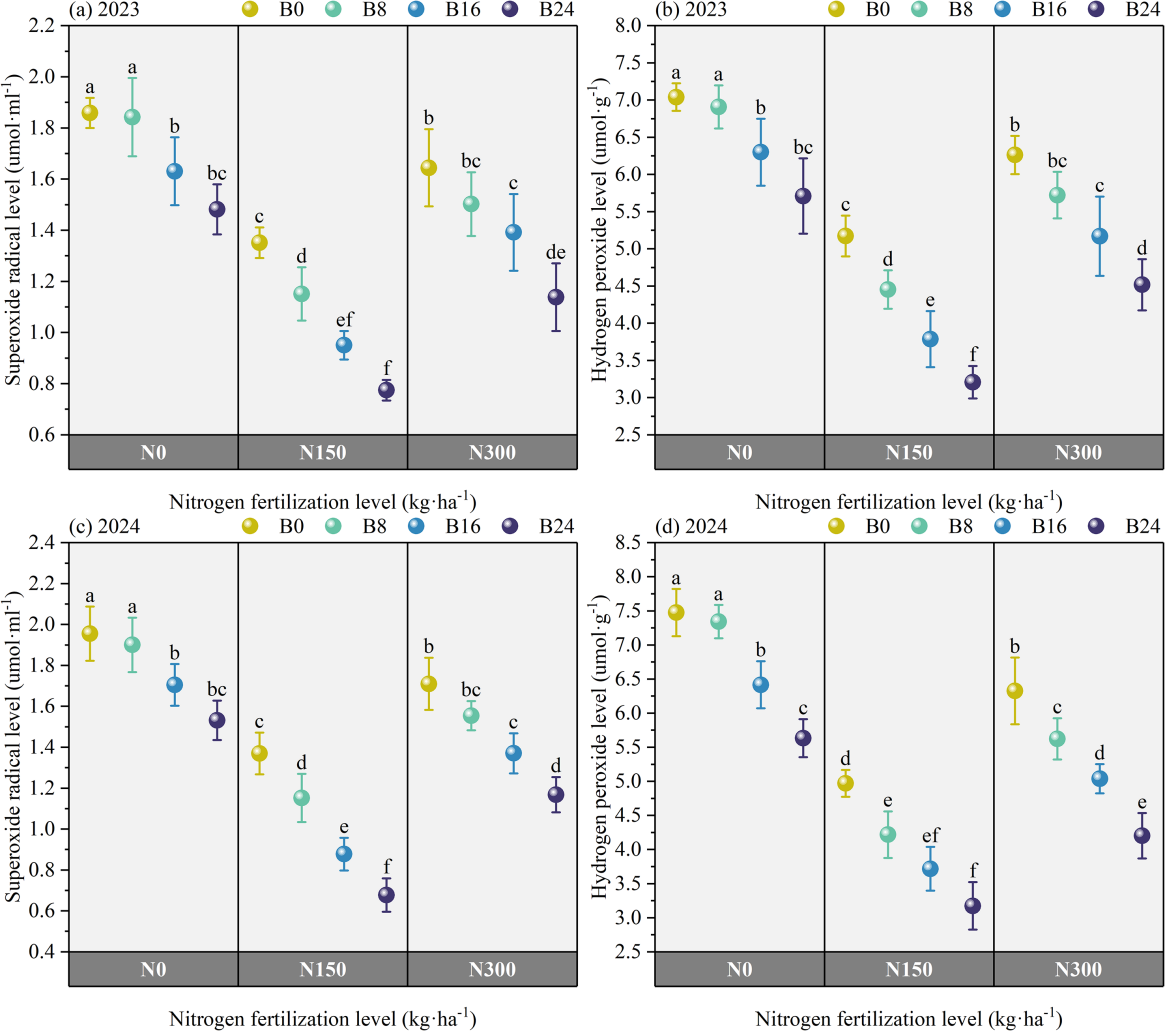
Effects of nitrogen fertilizer and aged biochar on H_2_O_2_ and O_2_^−^ levels. **(A)** superoxide radical level in 2023, **(B)** hydrogen peroxide in 2023; **(C)** superoxide radical level in 2024, **(D)** hydrogen peroxide in 2024.

**Figure 6** shows that the content of H_2_O_2_ and O_2_^−^ under N150 and N300 treatments significantly decreased by 56.20% and 19.78%, and 61.20% and 20.00% in 2023, and by 67.08% and 26.79%, and 74.00% and 22.20% in 2024, compared to N0. At the N150 level, compared to B0 treatment, the content of H_2_O_2_ and O_2_^−^ under B8, B16, and B24 treatments significantly decreased by 16.16%, 36.61%, and 61.30%, and 21.48%, 27.99%, and 74.95% in 2023, and by 17.83%, 33.74%, and 56.63%, and 11.23%, 35.14%, and 101.48% in 2024; respectively. These results indicate that combined nitrogen reduction and biochar amendment (1) improves superoxide dismutase-catalyzed conversion of O_2_^−^ to H_2_O_2_, and (2) promotes H_2_O_2_ scavenging via the AsA-GSH cycle, collectively reducing intracellular ROS levels and mitigating oxidative damage.

### 3.7 Effects of nitrogen fertilizer and aged biochar on non enzymatic antioxidant content

**Figure 7:**
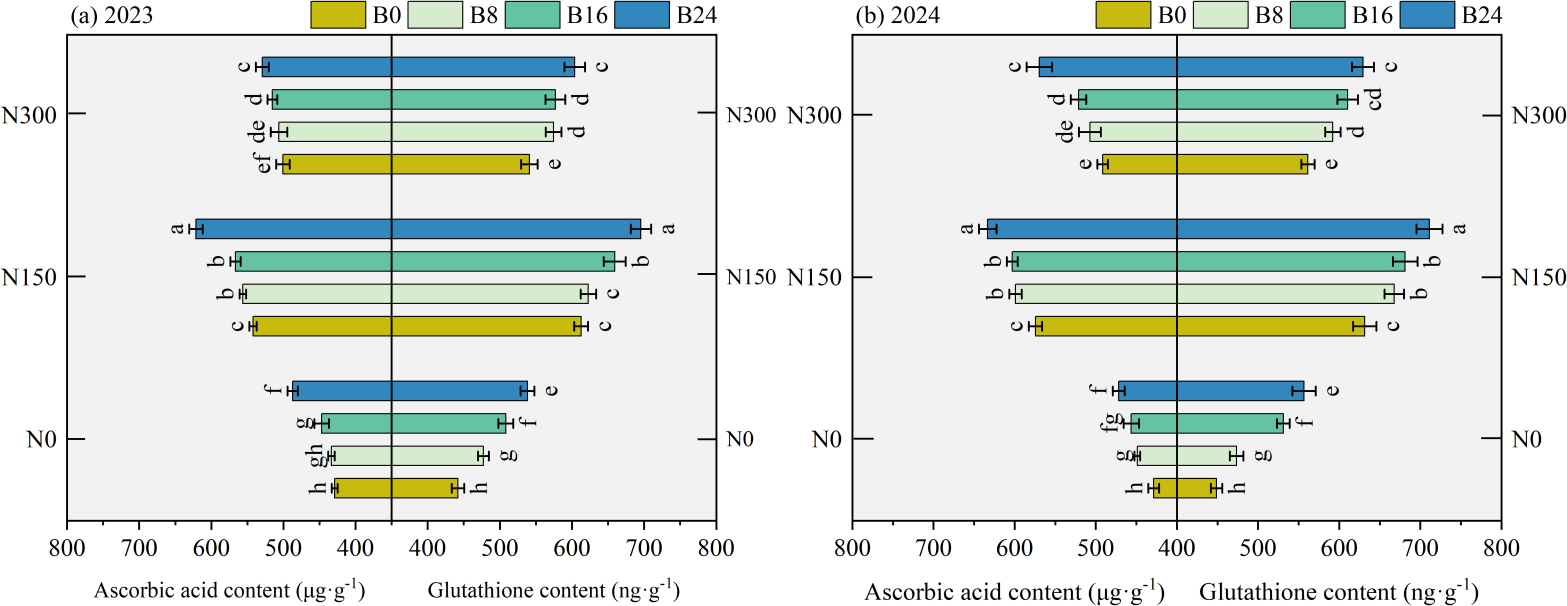
Effects of nitrogen fertilizer and aged biochar on AsA and GSH contents. **(A)** ascorbic acid and glutathione content in 2023, **(B)** ascorbic acid and glutathione content in 2024; AsA, ascorbic acid, GSH, glutathione.

As shown in **Figure 7**, compared to N0, N150 and N300 significantly increased AsA and GSH content by 27.79% and 14.18%, and 31.53% and 16.81% in 2023, and by 34.57% and 15.74%, and 33.58% and 19.06% in 2024. At the N150 level, compared to B0 treatment, the B8, B16, and B24 treatments significantly increased AsA and GSH content by 2.61%, 4.49%, and 14.60%, and 1.65%, 7.61%, and 13.54% in 2023, and by 4.30%, 4.96%, and 10.26%, and 5.77%, 7.89%, and 12.64% in 2024; respectively. These findings indicate that combined nitrogen reduction and biochar application improves both substrate availability and electron transfer capacity in the ascorbate-glutathione cycle, thereby accelerating hydrogen peroxide detoxification.

### 3.8 Effects of nitrogen fertilizer and aged biochar on antioxidant enzyme activity

As shown in **Table 3**, nitrogen fertilizer and biochar significantly affected the activities of SOD, APX, MDHAR, DHAR, and GR in both years (P<0.001). Compared to N0, N150 and N300 significantly increased SOD activity by 23.52% and 14.83%, APX activity by 37.05% and 19.88%, MDHAR activity by 18.57% and 8.96%, DHAR activity by 29.36% and 15.58%, and GR activity by 20.16% and 10.39% in 2023, similarly, in 2024, N150 and N300 increased SOD activity by 24.94% and 13.81%, APX activity by 32.78% and 18.73%, MDHAR activity by 29.17% and 14.80%, DHAR activity by 41.35% and 23.45%, and GR activity by 21.12% and 8.89%. At the N150 level, compared to B0, the B8, B16, and B24 significantly increased SOD activity by 4.28%, 6.96%, and 13.98%, APX activity by 4.71%, 8.62%, and 12.48%, MDHAR activity by 2.24%, 6.92%, and 11.81%, DHAR activity by 4.67%, 6.05%, and 11.76%, and GR activity by 3.09%, 5.21%, and 7.99% in 2023. Similarly, the B8, B16, and B24 treatments increased SOD activity by 1.30%, 5.31%, and 13.31%, APX activity by 2.60%, 6.30%, and 11.23%, MDHAR activity by 4.80%, 8.71%, and 14.45%, DHAR activity by 4.64%, 5.16%, and 11.24%, and GR activity by 3.68%, 7.35%, and 11.66% in 2024. These results illustrate that nitrogen reduction with biochar upregulates key enzymatic activities in the ascorbate-glutathione cycle, accelerating its overall reaction kinetics and thereby promoting more efficient hydrogen peroxide metabolism.

**Table 3:** Effects of nitrogen fertilizer and aged biochar on SOD, APX, MDHAR, DHAR and GR activities.

Year	Nitrogen level (kg·ha^-1^)	Biochar (t·ha^-1^)	SOD (U·g^-1^)	APX (U·g^-1^)	MDHAR (U·g^-1^)	DHAR (U·g^-1^)	GR (U·g^-1^)
2023	N0	B0	383.89±4.92h	559.45±8.69j	3.83±0.05h	13.33±0.14h	0.84±0.01h
		B8	401.13±6.46g	587.68±8.44i	4.08±0.05g	14.37±0.15g	0.87±0.01g
		B16	415.05±5.64f	617.48±13.12h	4.18±0.06fg	14.48±0.16g	0.87±0.01g
		B24	442.11±9.08e	652.95±14.10g	4.28±0.04ef	15.35±0.13f	0.91±0.01f
	N150	B0	477.03±6.23c	778.13±14.95d	4.61±0.12cd	17.61±0.13c	1.01±0.01d
		B8	497.47±3.68b	814.78±12.32c	4.71±0.16c	18.44±0.42b	1.04±0.01c
		B16	510.22±5.27b	845.17±13.60b	4.93±0.16b	18.68±0.30b	1.06±0.02b
		B24	543.72±11.79a	875.25±17.48a	5.15±0.18a	19.68±0.33a	1.09±0.02a
	N300	B0	435.72±5.01e	690.89±16.96f	4.29±0.06ef	15.47±0.14f	0.92±0.01f
		B8	462.44±4.7 3d	698.36±13.50f	4.46±0.04de	16.31±0.13e	0.95±0.01e
		B16	479.70±8.07c	733.94±10.49e	4.48±0.06d	17.11±0.16d	0.97±0.01e
		B24	507.86±10.48b	774.93±14.48d	4.61±0.10cd	17.60±0.14c	1.00±0.01d
	Interactions						
	Nitrogen		***	***	***	***	***
	error 1		17.37	579.20	0.00	0.02	0.00
	Biochar		***	***	***	***	***
	Nitrogen × Biochar		ns	ns	ns	ns	ns
	error 2		61.40	101.69	0.01	0.06	0.00
2024	N0	B0	376.08±6.85h	544.43±10.88j	3.71±0.09h	13.37±0.18h	0.85±0.01h
		B8	402.24±7.16g	574.14±8.30i	3.86±0.06h	14.42±0.15g	0.87±0.01g
		B16	412.10±6.57g	616.22±12.52h	4.08±0.05g	14.35±0.16g	0.88±0.01g
		B24	447.89±8.11ef	655.59±10.48g	4.36±0.09f	16.14±0.12f	0.91±0.01f
	N150	B0	487.47±4.89c	755.47±13.90cd	4.83±0.05d	19.56±0.18c	1.01±0.01d
		B8	493.79±10.23c	775.12±15.65c	5.07±0.15c	20.47±0.18b	1.04±0.01c
		B16	513.35±4.08b	803.10±13.86b	5.26±0.12b	20.57±0.14b	1.08±0.01b
		B24	552.34±8.23a	840.31±14.09a	5.53±0.11a	21.76±0.15a	1.13±0.01a
	N300	B0	438.17±10.24f	680.53±14.06f	4.38±0.08f	16.37±0.11f	0.92±0.01f
		B8	455.00±6.24e	682.85±11.57f	4.52±0.06ef	17.54±0.08e	0.95±0.01e
		B16	473.11±6.57d	724.39±11.62e	4.66±0.11e	18.59±0.11d	0.96±0.01e
		B24	498.33±7.60c	750.41±14.26d	4.83±0.12d	19.44±0.13c	1.00±0.01d
	Interactions						
	Nitrogen		***	***	***	***	***
	error 1		56.71	118.83	0.01	0.04	0.00
	Biochar		***	***	***	***	***
	Nitrogen × Biochar		ns	ns	ns	***	*
	error 2		52.91	183.15	0.10	0.02	0.00

ns, not significant, ^⁎^significant at P < 0.05, ^⁎⁎^significant at P < 0.01 and ^⁎⁎⁎^significant at P < 0.001. Error 1 is main plot error; error 2 is total error, numbers are mean square values. SOD, superoxide dismutase; APX, ascorbate peroxidase; MDHAR, monodehydroascorbate reductase; DHAR, dehydroascorbic reductase; and GR, glutathione reductase.

### 3.9 Effects of nitrogen fertilizer and aged biochar on grain yield

**Figure 8:**
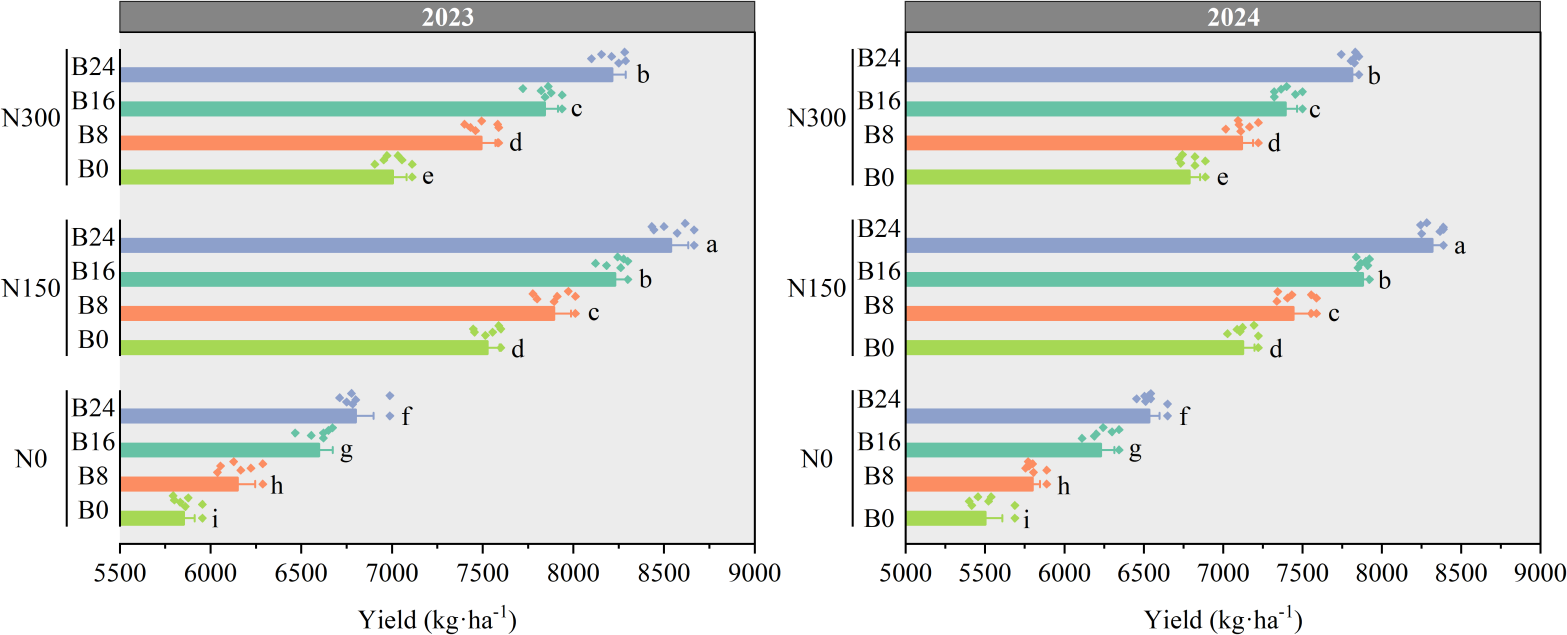
Effects of nitrogen fertilizer and aged biochar on grain yield.

As shown in **Figure 8**, grain yield of maize was significantly higher in the N150 treatment than in the N300 and N0 treatments in 2023 and 2024. Compared to N0, grain yield under N150 and N300 treatments significantly increased by 26.73% and 20.29%, and 27.85% and 20.97% in 2023 and 2024, respectively. At the N150 level, compared to B0 treatment, the B8, B16, and B24 treatments significantly increased grain yield by 4.88%, 9.36%, and 13.44% in 2023, and by 4.45%, 10.59%, and 16.74% in 2024.

### 3.10 Variance analysis of reactive oxygen species, antioxidants and yield

As shown in **Table 4**, Both nitrogen fertilizer and biochar, as well as their interactions, significantly affected the content of AsA, and GSH, along with grain yield in both years (P < 0.05). Significant effects on O_2_^−^ and H_2_O_2_ levels were observed with nitrogen fertilizer and biochar application (P < 0.001), whereas no significant effects on O_2_^−^ and H_2_O_2_ level were observed with their interactions (P > 0.05).

**Table 4:** Variance analysis of ROS, antioxidants and yield.

Year	Source	O_2_^−^	H_2_O_2_	AsA	GSH	Yield
2023	Nitrogen	***	***	***	***	***
	error 1	0.00	0.14	95.10	316.78	7233.94
	Biochar	***	***	***	***	***
	Nitrogen × Biochar	ns	ns	***	**	**
	error 2	0.02	0.13	34.21	93.84	6647.30
2024	Nitrogen	***	***	***	***	***
	error 1	0.01	0.10	141.11	90.45	2220.04
	Biochar	***	***	***	***	***
	Nitrogen × Biochar	ns	ns	**	*	**
	error 2	0.01	0.10	50.73	155.61	5827.94

ns, not significant, ^⁎^significant at P < 0.05, ^⁎⁎^significant at P < 0.01 and ^⁎⁎⁎^significant at P < 0.001. Error 1 is main plot error; error 2 is total error, numbers are mean square values. O_2_^−^, superoxide radical; H_2_O_2_, hydrogen peroxide; AsA, ascorbic acid; GSH, glutathione.

### 3.11 Correlation analysis of soil characteristics, root system characteristics, antioxidant enzymes and grain yield

**Figure 9:**
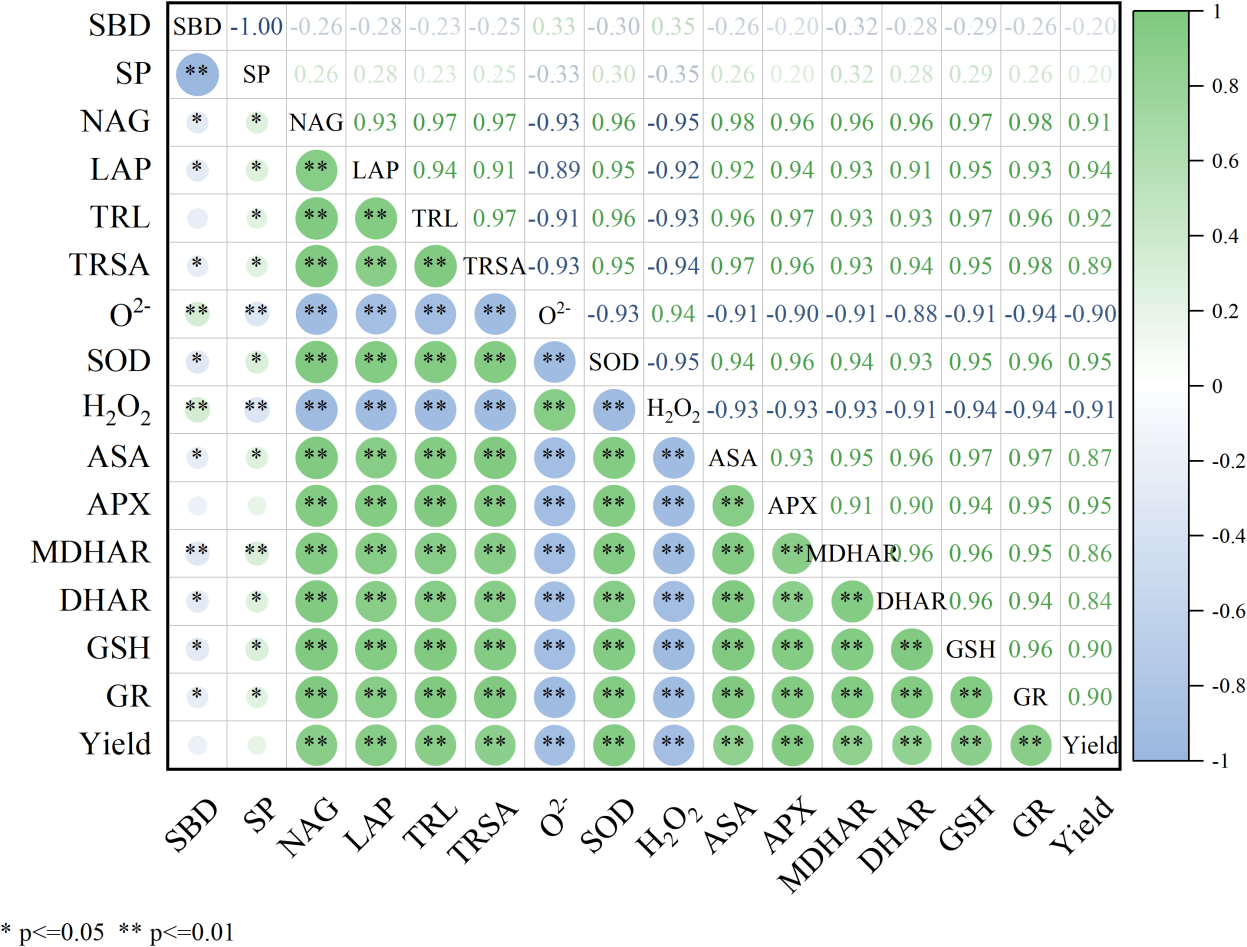
Correlation analysis of soil characteristics, root characteristics, antioxidant enzymes and yield (SBD, soil bulk density; SP, soil porosity; NAG, N-acetyl-β-D-glucosaminidase; LAP, leucine aminopeptidase; TRL, total root length; TRSA, total root surface area; SOD, superoxide dismutase; APX, ascorbate peroxidase; MDHAR, monodehydroascorbate reductase; DHAR, dehydroascorbic reductase; GR, glutathione reductase; ASA, ascorbic acid; GSH, glutathione; O_2_^−^, superoxide radical; H_2_O_2_, hydrogen peroxide).

As presented in **Figure 9**, SP was significantly and positively correlated with the activities of NAG (r=0.264) and LAP (r=0.275), TRL (r=0.232), and TRSA (r=0.254) (P<0.05), indicating that the application of nitrogen fertilizer and biochar significantly reduced the soil bulk density, increased porosity, and consequently improved soil microbial metabolism activity and nutrient availability, ultimately facilitating maize root system development. A highly significant positive correlation (P<0.01) was observed between maize root morphology (length: r=0.959, 0.970, and 0.947; surface area: r=0.947, 0.956, and 0.980) and the activities of key antioxidant enzymes (SOD, APX, GR), suggesting that root system development substantially enhances the maize enzymatic antioxidant capacity. Significant inverse correlations were observed between antioxidant enzymes (SOD: r=-0.930 and -0.953; APX: r=-0.904 and -0.933) and reactive oxygen species (O_2_^−^ and H_2_O_2_): (P < 0.01), while positive correlations were detected with yield (SOD: r = 0.953; APX: r = 0.951). These results demonstrate that elevated SOD and APX activities effectively scavenged ROS, thereby stabilizing crop productivity.

### 3.12 Path analysis

**Figure 10:**
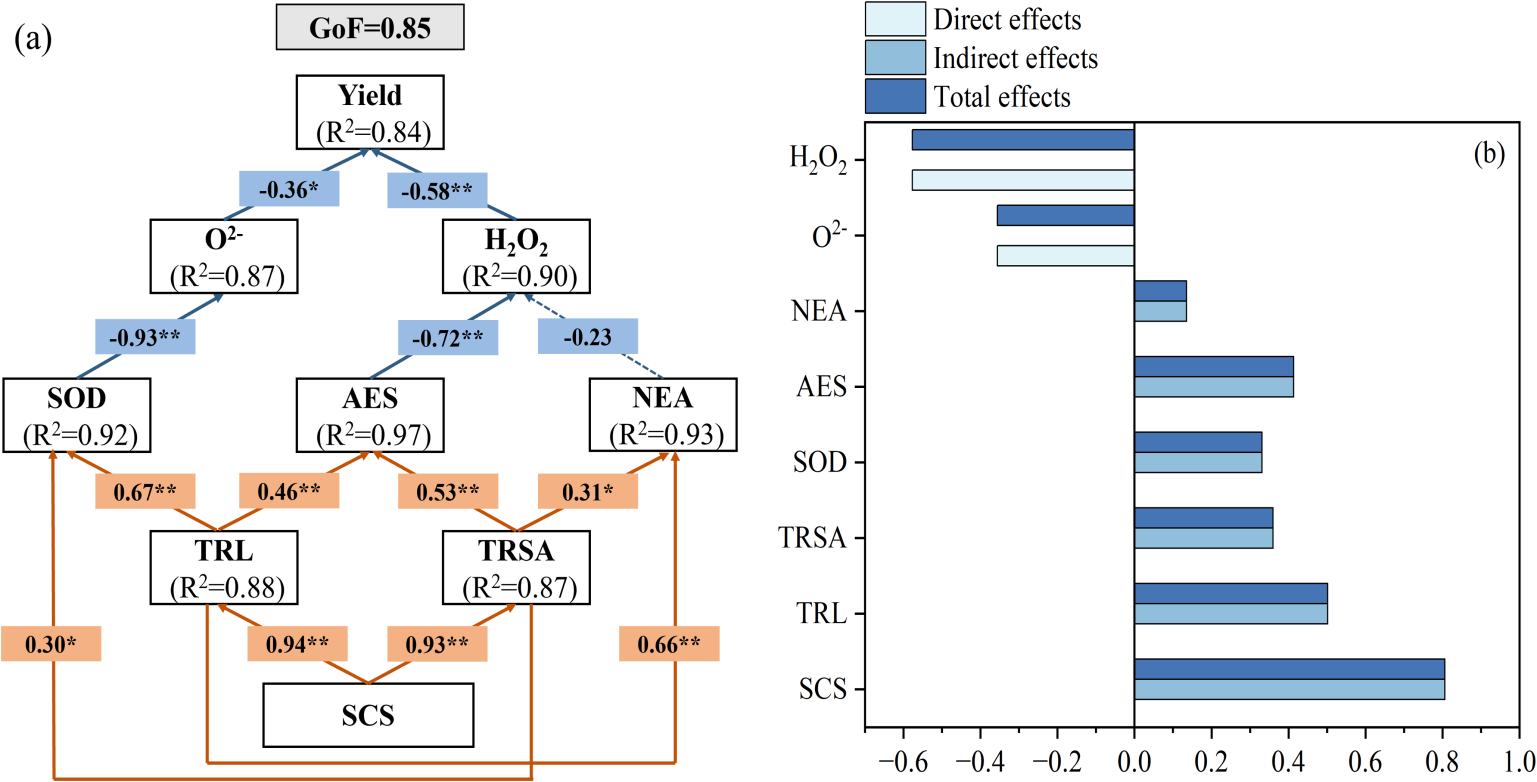
Path analysis of soil characteristics, root characteristics, ascorbic acid-glutathione cycle and yield. **(A)** Partial least squares model (PLS) revealed the direct and indirect effects of nitrogen and biochar on maize yield. **(B)** Standardized effects of SCS, TRL, TRSA, SOD, AES, NEA, O_2_-, and H_2_O_2_ on maize yield. B, standardized coefficient; SCS, soil characteristics, including soil bulk density, soil porosity, N-acetyl-β-D-glucosaminidase, and leucine aminopeptidase; TRL, total root length; TRSA, total root surface area; SOD: superoxide dismutase; AES, antioxidant enzymes, including ascorbate peroxidase, monodehydroascorbate reductase, dehydroascorbic reductase, and glutathione reductase; NEA, non enzymatic antioxidant, including ascorbic acid and glutathione.

A partial least squares (PLS) path model was employed to evaluate the relative contributions of soil properties, root system architecture, enzymatic antioxidants, and non-enzymatic antioxidants to grain yield (**Figure 10A**). Path analysis indicated that the path model exhibited a goodness-of-fit (GoF) of 0.85. Significant direct positive effects of soil properties were observed on both root length (β = 0.88, P < 0.01) and root surface area (β = 0.87, P < 0.01), as indicated by path coefficient analysis. Both TRL and TRSA showed significant positive direct effects on SOD, AES, and NEA (β = 0.67, 0.46 and 0.66, P < 0.01, and β = 0.30, 0.53, and 0.31, P < 0.05). A significant direct negative effect of SOD on O_2_^−^ was observed (β = -0.93, P < 0.01), while O_2_^−^ itself exerted a significant negative effect on grain yield (β = -0.36, P < 0.05). A significant direct negative effect of AES on H_2_O_2_ was observed (β = -0.72, P < 0.01), while H_2_O_2_ itself exerted a significant negative effect on yield (β = -0.58, P < 0.01) (**Figure 10B**). These findings reveal that soil property optimization directly enhances root system development, which subsequently upregulates antioxidant enzyme activities to mitigate reactive oxygen species (ROS) accumulation, thereby indirectly stabilizing grain yield.

## 4 Discussion

The application of nitrogen fertilizer and biochar significantly reduced soil bulk density and increased soil porosity. Furthermore, root system development was positively affected, with both root length and surface area showing significant expansion. Primary mechanisms by which biochar enhances root growth include its ability to modify soil physical properties. When incorporated into soil, biochar significantly reduces bulk density and compaction while improving porosity, water retention capacity, and gas permeability. These modifications collectively decrease mechanical impedance to root penetration and enhance the root system’s capacity for water and nutrient acquisition (Xiao et al., 2016). The study revealed that biochar application significantly decreased soil bulk density and compaction while concurrently increasing the relative proportions of both gaseous and liquid phases in the soil system. These structural modifications ultimately enhanced maize root development, as manifested by increased root length, total absorption area, and active absorption area (Sun et al., 2017; Liu et al., 2020). Secondly, biochar’s exceptional adsorption capacity and ion-exchange properties effectively mitigate soil nitrogen loss through multiple pathways: (1) reducing ammonia volatilization via surface adsorption, (2) decreasing nitrate leaching through ionic retention, (3) suppressing denitrification by improving soil aeration, and (4) minimizing erosion-induced loss through soil stabilization (Liu et al., 2019). Studies have demonstrated that biochar amendment significantly suppresses soil denitrification intensity and inhibits the microbial-mediated conversion of nitrate-nitrogen (NO_3_^−^-N) to nitrous oxide (N_2_O) (Li et al., 2021). Thirdly, biochar’s extensive specific surface area and highly porous architecture provide optimal microhabitats for soil microbiota. These physicochemical characteristics induce microbial community restructuring, stimulate metabolic activity, and enhance extracellular enzyme production, ultimately improving nutrient bioavailability (Pokharel et al., 2020). Biochar consists mainly of carbon and ash, with a carbon content of 70-80% (Lehmann and Joseph, 2009). Elevated biochar application rates induce microbial nitrogen immobilization through competitive fixation, leading to transient decreases in plant-available nitrogen pools. Conversely, supplemental nitrogen inputs provide essential substrates for nitrogen-cycling microbiota, stimulating their metabolic activity. This stimulation upregulates key nitrogen-metabolizing enzymes, thereby enhancing nitrogen transformation rates and improving overall nitrogen use efficiency (Ibrahim et al., 2020). The present study indicates that combined application of nitrogen fertilizer and biochar significantly increases soil N-acetyl-β-glucosaminidase (NAG) and leucine aminopeptidase (LAP) activities. These results are consistent with the findings of Zhang et al. (2019) and Zhang et al. (2021), who reported that nitrogen fertilizer and biochar co-application enhances urease, NAG, and LAP activities, thereby promoting nitrogen cycling in the soil-microbe-plant system. Ash contains multiple mineral components, such as K, Ca, Na, Mg, P, S, etc., which can be directly supplied to microorganisms and crop roots for absorption and utilization (Lehmann and Joseph, 2009).

Our results demonstrate that nitrogen application significantly enhanced antioxidant enzyme activities while reducing O_2_^−^ and H_2_O_2_ accumulation. Maize leaves exhibited more severe oxidative stress under high nitrogen fertilization (N300) compared to moderate nitrogen (N150), as indicated by elevated accumulation of both O_2_^−^ and H_2_O_2_ at higher nitrogen application rates. The elevated ROS levels observed under N300 fertilization may result from the following: (1) Under drought conditions, high nitrogen application can lead to luxury nitrogen uptake, increasing the demand for carbon skeletons and reducing equivalents. Constrained photosynthetic carbon fixation may cause a carbon–nitrogen imbalance, limiting the synthesis of antioxidant metabolites such as AsA and GSH and weakening ROS scavenging; (2) Luxury nitrogen uptake may result in NH_4_^+^ accumulation or disrupt the balance of essential ions such as K^+^ and Mg²^+^, directly promoting ROS overproduction and suppressing the activity of some antioxidant enzymes; (3) High nitrogen levels increase chlorophyll content and photosynthetic potential, but under drought-induced CO_2_ limitation, excess electrons can accumulate in the photosynthetic electron transport chain and leak to O_2_, enhancing ROS generation; (4) Nitrogen-induced canopy expansion elevates transpiration demand, which under limited water availability intensifies water deficit and restricts photosynthesis, contributing to higher ROS levels.

Abiotic and biotic stresses can induce the generation and accumulation of ROS, such as O_2_^−^, H_2_O_2_, and hydroxyl radical (OH·) (Kanazawa et al., 2000). ROS disrupt various cellular functions by damaging nucleic acids, oxidizing proteins, and inducing lipid peroxidation in cellular membranes (Noctor, 2005). Under stress conditions, plants trigger their inherent antioxidant defense systems through the coordination of enzyme activity and non-enzymatic components (Dumanovic et al., 2020). The SOD eliminates O_2_^−^ by catalyzing its dismutation, one O_2_^−^ being reduced to H_2_O_2_ (Garg and Manchanda, 2009). The APX-mediated reaction utilizes AsA as an electron donor to catalyze the conversion of H_2_O_2_ into H_2_O and O_2_ (Hasanuzzaman et al., 2021). Upon stress exposure, AsA-GSH cycle in plant cell activates to confer tolerance mechanisms in plants with key components such as DHA, GSH, GSSG, DHAR, MDHAR, and GR involved (Hasanuzzaman et al., 2023). Upon stress exposure, both AsA and GSH contents were increased in our observation. These results are in line with earlier findings Hannan et al. (2021) where it was explained that biochar facilitates plants tolerance capacity uplifting the bioactive compounds (protein, ascorbic acid, amino acid, carotenoid) under stressed conditions and ultimately can improve defense mechanisms of plants altering the AsA-GSH pool. Furthermore, the activities of SOD, APX, MDHAR, DHAR, and GR were increased after nitrogen and biochar application in our observation. Biochar amendments in soil can increase plant productivity by improving the physical, chemical, and biological properties of the soil (Asai et al., 2009; Graber et al., 2010). The beneficial effects of biochar application were attributed to elevated antioxidant enzyme activities and reduced O_2_^−^ and H_2_O_2_ concentrations. Previous studies have shown that biochar mitigates abiotic stresses on jute by stimulating non-enzymatic (AsA and GSH) and enzymatic (APX, MDHAR, DHAR, GR, SOD, and GSH-Px) activities to mitigate ROS damage (Hasanuzzaman et al., 2021). Gharred et al. (2022) experimented that biochar amendment increases water use efficiency under water deficit conditions, which in turn mediate non-enzymatic antioxidant (ASA and DHAsA) concentrations and enzymatic antioxidant (SOD, APX, GPOX, and GR) activities to buffer the adverse effects of drought on the photosynthetic apparatus. The favorable response was greater for 24t·ha^-1^ biochar relative to lower application rates in our study. However, Cong et al. (2023) reported that excessive fresh biochar application may inhibit maize growth and compromise antioxidant capacity during the initial application period. This inhibition could be due to multiple reasons. Firstly, the pH and CEC of biochar are high, and excessive biochar application to sandy loam soils (typically alkaline) will elevate soil pH and CEC beyond optimal crop growth thresholds, consequently impairing nutrient acquisition (Yuan and Xu, 2010); Secondly, the high-temperature pyrolysis process under oxygen-deficient conditions used to produce biochar may generate contaminants capable of causing both plant toxicity and cellular damage (Hu et al., 2021); And biochar may adversely impact the beneficial soil microbial communities (Mukherjee and Lal, 2014); Finally, fresh biochar due to its negatively charged surface can adsorb cationic nutrients (Yao et al., 2012), leading to their incomplete utilization by plants, which may negatively impact plant growth (Kammann et al., 2015). Inhibition of antioxidant enzyme activities by high biochar application was not observed in this investigation. This could be attributed to the aging of the biochar. Potential explanations include: (1) Natural aging processes may reduce biochar’s alkalinity through gradual pH decrease, consequently counteracting the soil pH increase caused by high-dose biochar amendments (Wang et al., 2020); (2) Natural aging processes may facilitate the accumulation of soil minerals on biochar surfaces, leading to the formation of organo-mineral complexes that occlude surface pores and fissures. This physical encapsulation could diminish the biochar’s adsorption capacity while concurrently enhancing nutrient bioavailability for plant uptake (Wang et al., 2021). (3) Biochar application rates in this study remained below the critical thresholds for both soil capacity and plant physiological tolerance. Khan et al. (2021) concluded that antioxidant enzyme activities and osmoregulatory substance contents were suppressed at biochar application rates above 30 t·ha^-1^; (4) It is also worth noting that the biochar amended soil may have suffered a dilution effect due to conventional tillage and long-term field management operations during the eight-year period.

Multiple studies conducted in different regions of the world have found that the biochar supplementation may produce different results, which are closely related to raw materials, pyrolysis temperature, soil properties, crop type, farming methods, and climate (Jeffery et al., 2016). Therefore, further research is needed to verify and optimize the technology of biochar application, to achieve its potential benefits on plant growth and yield.

## 5 Conclusion

In conclusion, nitrogen application promoted root growth, antioxidant enzymes, and lowered ROS levels in dryland maize versus non-nitrogen application. N150 nitrogen significantly increased soil nitrogen metabolizing enzyme activities, root growth, and AsA-GSH cycle activities while decreasing ROS accumulation in maize, as compared to N300 nitrogen. Biochar applied to soil decreased soil bulk density, enhanced porosity, and stimulated NAG and LAP activities in comparison with the non-biochar treatment. Thus, the application of aged biochar, particularly at 24t·ha^-1^, markedly increased total root length and root surface area, improved the content of AsA and GSH, and enhanced the activities of SOD, APX, MDHAR, DHAR and GR while decreasing O_2_^−^ and H_2_O_2_ in the plants. Moreover, we have demonstrated differences in response of soil properties, root characteristics, and antioxidant activities to different nitrogen and biochar levels under dryland maize cultivation systems. The presented results supported the view that co-application of nitrogen and biochar enhanced soil physicochemical properties and stimulated root growth, subsequently intensifying the AsA-GSH cycle to alleviate oxidative stress caused by O_2_^−^ and H_2_O_2_ in dryland maize. Based on these modified effects of nitrogen and biochar in the soil, the N150B24 combination is recommended as the optimal agronomic management practice to enhance soil properties, promote root development, and improve drought tolerance of maize under dryland cultivation.

## Data Availability

The raw data supporting the conclusions of this article will be made available by the authors, without undue reservation.
